# Euthanasia of animals – association with veterinarians’ suicidal thoughts and attitudes towards assisted dying in humans: a nationwide cross-sectional survey (the NORVET study)

**DOI:** 10.1186/s12888-023-05402-7

**Published:** 2024-01-02

**Authors:** Helene Seljenes Dalum, Reidar Tyssen, Torbjørn Moum, Magne Thoresen, Erlend Hem

**Affiliations:** 1 Department of Behavioural Medicine, Institute of Basic Medical Sciences, Faculty of Medicine, University of Oslo, Blindern, P.O. Box 1111, NO-0317 Oslo, Norway; 2 Institute for Studies of the Medical Profession, Oslo, Norway; 3 Department of Biostatistics, Institute of Basic Medical Sciences, Faculty of Medicine, University of Oslo, Oslo, Norway

**Keywords:** Veterinarians, Suicidal thoughts, Euthanasia, Assisted dying

## Abstract

**Background:**

Veterinarians are an occupational group with an increased suicide risk. Euthanasing animals may influence both veterinarians’ views on assisted dying in humans and their suicide risk. We investigated (I) attitudes towards assisted dying, (II) whether the field of work and the frequency of euthanasing animals were associated with positive attitudes towards human euthanasia, and (III) whether frequently euthanasing animals was associated with serious suicidal thoughts.

**Methods:**

We conducted a nationwide cross-sectional study among veterinarians in Norway (response rate: 75%). Logistic regression models were used to calculate the odds ratios for both positive attitudes towards human euthanasia and serious suicidal thoughts. The analyses were adjusted for socio-demographic and work-related factors.

**Results:**

Fifty-five percent of the veterinarians agreed that euthanasia should be permitted for humans with a fatal disease and short life expectancy. Working with companion animals was independently associated with positive attitudes towards human euthanasia (OR = 1.66 (95% CI: 1.23–2.23)), while veterinarians’ frequency of euthanasing animals was not. Frequency of euthanasing animals was independently associated with serious suicidal thoughts, OR = 2.56 (95% CI: 1.35–4.87).

**Conclusions:**

Veterinarians’ attitudes towards assisted dying in humans did not differ from those of the general population. Veterinarians’ frequency of euthanasing animals was not associated with positive attitudes towards euthanasia in humans. However, veterinarians working in companion animal practices were more likely to have positive attitudes towards euthanasia in humans. Moreover, euthanising animals five times or more a week was associated with serious suicidal thoughts. We need more research to infer about causality in these findings.

**Supplementary Information:**

The online version contains supplementary material available at 10.1186/s12888-023-05402-7.

## Background

**Fig. 1:**
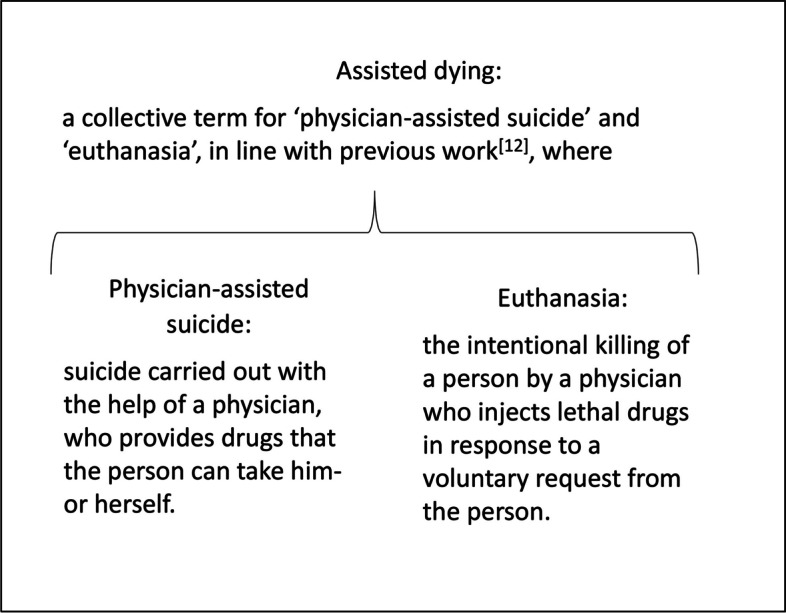
Definitions of assisted dying, physician-assisted suicide, and euthanasia

Several studies have found that veterinarians have higher suicide rates than the general population [1–3]. The reasons for elevated suicide risk among veterinarians remain unclear, and more studies exploring psychological and work-related risk factors are needed. Euthanasing animals is a task unique to veterinarians. It has been hypothesised that euthanasing animals may influence veterinarians’ view of assisted dying in humans [4] (see Fig. 1 for a definition of the term ‘assisted dying’) and veterinarians’ suicide risk [4, 5]. Studies on both of these possible influences are scarce.

A possible association between animal euthanasia and attitudes towards assisted dying in humans among veterinarians is scarcely studied. Previous studies have suggested that animal euthanasia might affect veterinarians' attitudes towards assisted dying, viewing euthanasia as a way to alleviate suffering through death [6], and even to alleviate their own suffering if they experience suicidal thoughts [4]. To our knowledge, only one study from the UK has investigated the possible association between euthanasing animals and being in favour of human euthanasia [7]. No such association was observed. However, the cited study included only veterinary students and recent graduates (32% response rate). In humans, knowledge of assisted dying through work experience is hypothesised to be associated with restrictive attitudes towards such practices [8, 9]. Several studies have indeed found that physicians have a more restrictive view on assisted dying than the general population [10–12]. This restrictive view is probably partly due to assisted dying being a dilemma, both in terms of professional value and role conflicts [12]. Veterinarians constitute an occupational group with extensive knowledge and experience of euthanasia of their animal patients. Similar to the conflicting role of physicians in end-of-life care, animal euthanasia has been called the ‘caring-killing paradox’, i.e. the conflicting situation of euthanasing animals when you are trained to provide care [13]. One study among veterinarians found that they have a more liberal view of assisted dying than physicians, thus questioning the hypothesis that knowledge of assisted dying is unambiguously associated with restrictive attitudes [9]. Moreover, palliative care and animal hospices are emerging in veterinary medicine [14]. The influence that animal euthanasia might have on veterinarians’ views on assisted dying in humans is scarcely studied. To date, little is known about the factors associated with the apparently more liberal attitudes among veterinarians towards assisted dying in humans, and whether attitudes towards euthanasia in humans is associated with having serious suicidal thoughts.

Veterinarians may experience several challenges with euthanasing animals, both in decision-making and ethical considerations, and this could be a source of moral stress [15]. These challenges may be partly due to major developments in veterinary medicine and the fact that companion animals are often considered family members. Most studies investigating the impact of euthanasing animals have been conducted on companion animal veterinarians. Thus, the impact of euthanising other animal species such as food-producing animals is not well known.

The three job stress factors—emotional demands, work-life balance and fear of complaints/criticism, and field of work—were not independently associated with serious suicidal thoughts among veterinarians in a previous study using the same sample of veterinarians as used in the present paper [16]. Nevertheless, veterinarians reported work factors as contributing to their suicidal thoughts. We suggested that there might be specific work factors for veterinarians that were not captured by the variables in the regression model used [16]. In the present paper, we therefore aimed to further investigate a work factor unique to veterinarians, namely animal euthanasia, and its possible association with both attitudes towards assisted dying and serious suicidal thoughts.

One study among veterinarians found a positive correlation between attitudes towards human euthanasia and an accepting attitude towards human suicide [7]. Moreover, an association between animal euthanasia and a lack of fear of death among veterinary students has been demonstrated [17]. Among graduate veterinarians, lower distress towards euthanasing animals was associated with a lesser fear of death, in line with findings among veterinary students [18]. Conversely, frequently euthanising animals was shown to attenuate the impact of depression on suicide risk, suggesting that euthanasia may be a protective factor against suicide [19]. The aforementioned study was cross-sectional, hindering any conclusions about causality in their findings. However, the authors suggested three possible explanations for a protective effect of euthanasia of animals on suicide risk: 1) veterinarians experience the impact that death has upon loved ones (animal owners), 2) euthanasia highlights the finality of death, and 3) emotional transference through repeated exposure to gratitude and thankfulness from animal owners may buffer the negative affect of depression [19]. Self-poisoning is a common method of suicide among veterinarians [1, 2, 5, 20–22]. Barbiturates (the class of drug used for euthanasing animals) have been shown to be the most commonly used drug for self-poisoning [2, 23]. Therefore, we need to further explore the role of euthanasing animals in relation to veterinarians’ suicidal thoughts. Also, when investigating a possible association between animal euthanasia and serious suicidal thoughts, it is important to control for possible confounders, such as working hours, and perceived colleague support. Working hours could be a confounder in the possible association between suicidal thoughts [24], and the frequency of animal euthanasia (more working hours could lead to a higher number of euthanasia procedures). Colleague support could be a confounder in the possible association between suicidal thoughts [25] and veterinarians’ frequency of euthanasing animals (lack of colleague support may lead to more euthanasia, as there is no one to consult).

As mentioned, veterinarians are a high-risk occupational group for suicide. The possible association between animal euthanasia and attitudes towards assisted dying in humans and between animal euthanasia and suicidal thoughts is scarcely studied among veterinarians. Research in representative samples of veterinarians in regards to both of these aspects, and their possible interplay, is lacking. Such research might contribute to a better understanding of the complexity of suicidality among veterinarians.

To our knowledge, this is the first nationwide study with a large representative sample of veterinarians investigating whether the main field of work and frequency of euthanasing animals is associated with positive attitudes towards human euthanasia, and whether the frequency of euthanasing animals is associated with serious suicidal thoughts among veterinarians. We would expect a significantly positive association both in research questions 2 and 3.

The research questions were as follows:

1. What are veterinarians’ attitudes towards physician-assisted suicide and euthanasia in humans?
2. Is the main field of work and veterinarians’ frequency of euthanasing animals associated with positive attitudes towards euthanasia in humans?
3. Is veterinarians’ frequency of euthanasing animals associated with serious suicidal thoughts?

## Methods

The sample included all veterinarians in Norway, holding valid authorisation in May 2020 (*n* = 4256), according to information retrieved from the Norwegian Food Safety Authority, which is the national authority granting veterinary authorisation. We excluded some veterinarians based on the following criteria: no residential address in Norway (*n* = 527), unknown current address (*n* = 196), working abroad (*n* = 62), and those deceased (*n* = 7), resulting in an eligible sample of 3464 participants.

A 12-page questionnaire, together with an information sheet, and a pre-paid reply envelope were distributed by mail to 3464 veterinarians in November 2020, with two reminders being sent in January and February. The questionnaire was in Norwegian, and it took between 15–30 min to complete. There was also a reminder in The Norwegian Veterinary Journal and on the webpage of the Norwegian Veterinary Association. Information about the survey was also disseminated through social media, both by the first author and by the Norwegian Veterinary Association. Five gift cards from a sports shop were drawn among the respondents of the survey to increase the response rate. The researchers did not know the identities of the participants.

This study was approved by the Regional Committees for Medical Research Ethics South East Norway (132704) and the Norwegian Centre for Research Data (674793).

## Instruments

### Dependent variable – research questions 1 and 2

Attitudes towards assisted dying were investigated using four statements previously used on physicians in Norway [12], namely: 1) ‘Physician-assisted suicide should be permitted for persons suffering from a fatal disease with a short remaining life expectancy’; 2) ‘Euthanasia should be permitted for persons suffering from a fatal disease with a short remaining life expectancy’; 3) ‘Assisted dying should be permitted also for persons suffering from an incurable chronic disease, but who are not dying’; and 4) ‘There are cases in which it may be right/morally defensible for the doctor to provide assisted dying, even though it is illegal’. The veterinarians were asked to state their agreement, on a Likert scale from 1–5, ranging from ‘strongly agree’ to ‘strongly disagree’. For research question 1, we investigated the level of agreement with each statement. Definitions of the terms assisted dying, physician-assisted suicide and euthanasia were included in the questionnaire.

Veterinarians routinely perform animal euthanasia, while prescription of medicinal products that animal owners can give to their animals is not common practice (in which the latter would be the parallel to physician-assisted suicide in humans). Therefore, we chose the second statement regarding attitudes towards euthanasia in humans as the dependent variable for research question 2, as this would be the procedure veterinarians have the most experience with through their work. The variable was dichotomised so that ‘agreed’ included both ‘strongly agree’ and ‘partially agree’, to make clear the distinction of those having positive attitudes towards the statement. ‘Disagree’ encompasses the remaining alternative responses (‘neither agree, nor disagree’, ‘partially disagree’ and ‘strongly disagree)’, in line with a previous study [12].

### Dependent variable – research question 3

Paykel’s questionnaire is a five-item instrument developed to study suicidal thoughts and attempts. The five items represent increasing severity, from unspecific suicidal feelings, and a wish to die, to suicidal thoughts, serious suicidal thoughts (plans), and suicide attempt [26]. Serious suicidal thoughts were used as the dependent variable for research question 3. The question was a slightly modified version of the fourth question of Paykel’s questionnaire [24, 26]: ‘Have you ever during the last year reached the point where you seriously considered taking your life, and even made plans how you would go about doing it?’ The responses were ‘never’, ‘hardly ever’, ‘sometimes’, and ‘often’. Responses were dichotomised into ‘never’ and ‘any frequency’, in line with Paykel’s original work. Paykel's question number four has been used as the dependent variable in several studies across multiple professions in Norway previously, including veterinarians [16, 27, 28].

### Exposure variables

The participants reported the following as their main fields of work: ‘companion animal practice’, ‘production animal practice’, ‘mixed clinical practice’, ‘equine practice’, ‘aquaculture’, ‘public administration’, ‘academia/research’, ‘pensioners’, and ‘other’ [16]. In the regression analyses, ‘mixed clinical practice’ was chosen as the reference category, as this could be viewed as the most traditional veterinary work in Norway. Since work-related factors were included in the model, pensioners were excluded from the regression analyses. Field of work was used as an exposure variable for research questions 2 and 3.

Frequency of euthanasing animals was measured using a single item: ‘If you work in clinical practice, how many euthanasia procedures do you perform on average in a normal week?’ Responses were categorised as follows; 0–4, 5–9, 10–14, and 15 or more. Due to low numbers in the latter three categories, the variable was dichotomised into 0–4/week and 5 or more/week. The total proportion of missing data on this instrument was 36%. This is probably due to the question being directed to veterinarians working in clinical practice. In our sample, however, many veterinarians work in non-clinical fields. After cross-tabulating the frequency of euthanasia with the different fields of work, it was evident that the majority of the missing answers to this question was among veterinarians in non-clinical positions, with a missing rate of 73% in aquaculture, 90% in public administration, 72% in academia/research, and 78% in other fields. Most veterinarians in these fields do not routinely perform euthanasia. Therefore, this lack of response was recoded to category ‘0–4’, as this seemed to be the most likely reason for the high proportion of missing answers in these fields of work. After recoding, the proportion of missing data was 2.9% for this instrument, which is acceptable. Veterinarians’ frequency of euthanasing animals was used as an exposure variable in research questions 2 and 3.

### Confounders

The use of age intervals was encouraged by The Norwegian Centre for Research Data to keep collected data as unidentifiable as possible. Age was therefore reported in the following intervals: 20–25, 26–30 (…) up to 66–70, and > 70 years old. These age categories were entered as continuous variables in the regression models. Marital status was dichotomised into married/cohabitant and single/divorced/separated/widowed. Gender, age, and marital status were included as possible confounders in research questions 2 and 3.

In addition to gender, age, and marital status, the self-reported average number of working hours per week was used as a measure of workload in research question 3.

Attitudes towards euthanasia in humans could be a possible confounder for serious suicidal thoughts, as well as for euthanasia of animals. A previous study among veterinarians found a significant positive correlation between attitudes towards human euthanasia and suicide [7]. Although scarcely studied, veterinarians’ positive attitudes towards euthanasia in humans may also influence attitudes towards animal euthanasia, possibly leading to a higher frequency of euthanasia of animals compared to the frequency among those with a negative attitude towards human euthanasia. Therefore, attitudes towards euthanasia in humans were included as a possible confounder in research question 3.

Colleague support was measured using the mean of two questions: ‘To what degree do you enjoy working with your colleagues?’ and ‘To what degree are you taken care of by your colleagues?’. Responses were given on a scale from 1 (not at all) to 7 (to a very high degree), as in previous studies [29, 30]. Cronbach’s α for the two items on colleague support in our sample was 0.84. The mean scores of the two questions on colleague support were used in the regression analyses for research question 3.

### Demographics

We received 2596 responses from 3464 participants (response rate: 75%). The most frequently reported age category was 41 – 45 years. Age varied between genders, with a higher proportion of younger women on the women’s side, and 65% of the men being over 50 years of age. In total, 70% of the participants were female and 30% were male, which is an accurate reflection of the gender distribution of the total population of veterinarians in Norway (personal communication, Bente N. Reve, The Norwegian Food Safety Authority, 12 July 2021). The age distribution in our sample had only minor differences from the target population, according to The Norwegian Directorate of Health, which has an overview of all registered health personnel in Norway. Additionally, rough estimates of the distribution between different fields of work in the NORVET study sample coincide with the major speciality associations in the Norwegian Veterinary Association, indicating that our sample is fairly representative concerning the distribution of fields of work as well. More details about the representativeness of the sample can be found in the doctoral thesis by Dalum [31].

A total of 139 (5%) veterinarians reported serious suicidal thoughts, a finding described in a past study [16].

### Statistical analyses

StataSE 17 was used for statistical analyses. The χ^2^ test was used to test for difference by gender. Bivariate and multivariable logistic regression models were used to estimate the odds ratios (OR) for the associations between exposure and dependent variables. Initially, all variables were analysed bivariately with the dependent variable (crude OR).

Goodness-of-fit was tested by Pearson’s goodness-of-fit test, and found satisfactory in all of the logistic regression models. The level of significance was set at 5% (*p* < 0.05). Two-way interaction terms between gender and the independent variables were entered to investigate gender-specific effects, with the main effect included in the model. Interaction terms were entered individually. Overall, missing data across our dependent and independent variables was low (less than 4%). A missing-data analysis was performed for each of the independent variables in both regression models. With the exception of the high prevalence of missing data on the item on frequency of euthanasing animals (see above), no other missing data trends were found.

## Results

Table 1 provides a description of the sample.

**Table 1:** Description of the independent variables in the present sample

Variable	Frequency (%) or mean value (SD)
**Gender (*n* = 2552)**	
Female	1776 (70%)
Male	776 (30%)
**Age**^a^**(*n* = 2547)**	
20–30	274 (11%)
31–40	697 (27%)
41–50	667 (26%)
51–60	432 (17%)
61–70	318 (13%)
> 70	159 (6%)
**Marital status (*n* = 2514)**	
Married/cohabiting	1962 (78%)
Single/divorced/widowed	552 (22%)
**Main field of work (*n* = 2522)**	
Companion animal practice	802 (32%)
Public administration	402 (16%)
Mixed clinical practice	268 (10%)
Academia/research	202 (8%)
Production animal practice	177 (7%)
Aquaculture	121 (5%)
Equine practice	102 (4%)
Other	250 (10%)
Pensioner	198 (8%)
**Working hours (range 0–99) (*n* = 2293)**	Mean 41.6 (SD = 12.07)
**Serious suicidal thoughts (*n* = 139/2562)**	139 (5.4%)
**Colleague support (range 1–7) (*n* = 2403)**	Mean 5.29 (SD = 1.38)
**Frequency of performance of euthanasia (*n* = 2350)**	
0–4/week	2233 (95%)
5 or more/week	117 (5%)

^a^Age was reported in five-year categories. In Table 1, age distribution is showed in 10-year categories, to improve readability

Of the veterinarians in this study, 63% strongly agreed or partially agreed with the statement, ‘Physician-assisted suicide should be permitted for persons suffering from a fatal disease with a short remaining life expectancy’. A total 55% strongly or partially agreed that ‘Euthanasia should be permitted for persons suffering from a fatal disease with a short remaining life expectancy’ and 51% strongly or partially agreed with the statement ‘There are cases in which it may be right/morally defensible for the doctor to provide assisted dying, even though it is illegal’. The statement, ‘Assisted dying should be permitted also for persons suffering from an incurable chronic disease, but who are not dying’ had the lowest consensus (43%) (Table 2).

**Table 2:** Veterinarians’ attitudes towards physician-assisted suicide and euthanasia in humans

Statement	Strongly agree, n (%)	Partially agree, n (%)	Neither agree, nor disagree, n (%)	Partially disagree, n (%)	Strongly disagree, n (%)	Total, n
1. ‘Physician-assisted suicide should be permitted for persons suffering from a fatal disease with a short remaining life expectancy.’^a^	805 (31.5%)	814 (31.9%)	318 (12.5%)	198 (7.8%)	420 (16.4%)	2555
2. ‘Euthanasia should be permitted for persons suffering from a fatal disease with a short remaining life expectancy.’^a^	666 (26.0%)	738 (28.9%)	399 (15.6%)	252 (9.9%)	500 (19.6%)	2555
3. ‘Assisted dying should be permitted also for persons suffering from an incurable chronic disease, but who are not dying.’^a^	414 (16.3%)	690 (27.1%)	545 (21.4%)	309 (12.1%)	590 (23.2%)	2548
4. ‘There are cases in which it may be right/morally defensible for the doctor to provide assisted dying, even though it is illegal.’^a^	525 (20.6%)	787 (30.8%)	432 (16.9%)	243 (9.5%)	568 (22.2%)	2555

^a^The prevalence of positive attitudes was significantly higher among female veterinarians compared to their male colleagues in all four statements

Agreement with all four questions was significantly higher among females than among males (Additional Table 1 – Veterinarians’ agreement on assisted dying by gender).

Working in companion animal practice was independently associated with an increased likelihood of positive attitudes towards euthanasia in humans in the multivariable model (Table 3), with companion animal practitioners having 1.66 higher odds of holding positive attitudes towards euthanasia in humans compared to veterinarians working in mixed clinical practice. Veterinarians’ frequency of euthanasing animals was not independently associated with positive attitudes towards euthanasia in humans. Being younger and being single were also significantly associated with positive attitudes towards euthanasia in humans. We found a significant interaction between gender and marital status (OR = 1.94, 95% CI 1.18 – 3.23), indicating that single women have more positive attitudes towards euthanasia in humans than women with a partner.

**Table 3:** Variables associated with positive attitudes towards human euthanasia

	Crude		Adjusted	
	OR	95% CI	OR	95% CI
Female (ref. = male)	1.46^*^	1.21 – 1.75	1.02	0.82 – 1.26
Age	0.86^*^	0.83 – 0.89	0.87^*^	0.83 – 0.91
Single (ref. = having a partner)	1.38^*^	1.13 – 1.69	1.35^*^	1.10 – 1.67
**Main field of work (ref. = mixed clinical practice)**				
Companion animals	1.69^*^	1.27 – 2.23	1.66^*^	1.23 – 2.23
Production animals	0.85	0.58 – 1.25	1.04	0.69 – 1.56
Equine practice	1.16	0.73 – 1.83	1.29	0.80 – 2.09
Aquaculture	1.10	0.71 – 1.69	1.07	0.69 – 1.67
Public administration	0.83	0.61 – 1.14	0.98	0.70 – 1.35
Academia/research	1.15	0.79 – 1.66	1.35	0.92 – 1.98
Other	0.96	0.68 – 1.36	1.07	0.74 – 1.53
Frequency of euthanasia (ref. 0–4/week)				
5 or more/week	1.42	0.95 – 2.11	1.32	0.88 – 2.00

*N* = 2222

^*^p < 0.05

Performing euthanasia more than five times per week was independently associated with a higher likelihood of serious suicidal thoughts in the multivariable model (Table 4). Veterinarians performing euthanasia five or more times per week had 2.56 higher odds of having serious suicidal thoughts than those performing euthanasia four or fewer times per week. Being single, having a positive attitude towards human euthanasia, and low perceived colleague support were also significantly associated with a higher likelihood of serious suicidal thoughts. No interaction with gender was found.

**Table 4:** Variables associated with serious suicidal thoughts

	Crude		Adjusted	
	OR	95% CI	OR	95% CI
Female (ref. = male)	1.55	0.999 – 2.401	1.30	0.78 – 2.18
Age	0.93	0.86 – 1.00	0.95	0.86 – 1.04
Single (ref. = having a partner)	2.38^*^	1.65 – 3.43	2.11^*^	1.42 – 3.15
Positive attitudes towards euthanasia in humans (ref. = disagree/indecisive)	2.68^*^	1.79 – 4.02	2.00^*^	1.29 – 3.09
Working hours	1.00	0.99 – 1.02	1.00	0.99 – 1.02
**Main field of work (ref. = mixed clinical practice)**				
Companion animals	1.38	0.74 – 2.57	1.39	0.69 – 2.81
Production animals	1.28	0.56 – 2.94	1.57	0.62 – 3.95
Equine practice	1.21	0.45 – 3.28	1.40	0.48 – 4.07
Aquaculture	1.01	0.37 – 2.73	1.20	0.42 – 3.42
Public administration	1.08	0.53 – 2.20	1.43	0.63 – 3.21
Academia/research	1.12	0.49 – 2.56	1.24	0.49 – 3.11
Other	0.82	0.35 – 1.91	0.88	0.34 – 2.33
Colleague support	0.64^*^	0.57 – 0.71	0.63^*^	0.56 – 0.72
Frequency of euthanasia (ref. 0–4/week)				
5 or more/week	2.48^*^	1.38 – 4.46	2.56^*^	1.35 – 4.87

*N* = 2083

^*^p < 0.05

As there were differences in the relative prevalence of frequency of euthanasia between the different fields of work, we cross-tabulated the frequency of euthanasia with field of work. This cross-table can be found as an additional file (Additional Table 2 – Cross-table frequency of euthanasia of animals and main field of work).

## Discussion

A major finding of this study is that veterinarians’ views on assisted dying in humans do not differ from those of the general population in Norway [32]. Working in companion animal practices was independently associated with positive attitudes towards euthanasia in humans, while veterinarians’ frequency of euthanasing animals was not. Performing euthanasia more than five times per week was independently associated with serious suicidal thoughts compared to those performing euthanasia four or fewer times per week.

Knowledge regarding euthanasia acquired by veterinarians through their work does not seem to lead to a restrictive view of assisted dying in humans. This is in contrast to physicians, where restrictive attitudes towards assisted dying in humans have been shown in several studies [9, 12]. In our study, veterinarians had attitudes towards assisted dying comparable to those of the general population [32], and more liberal attitudes than physicians [12]. This trend is in line with a similar study in Sweden that included veterinarians, physicians, and the general population [9]. Overall, the prevalence of positive attitudes was significantly higher among female veterinarians compared to their male colleagues, however, no gender difference was found in the multivariable model. This is in line with a previous study, where no gender difference in attitudes towards assisted dying was found [9]. The reasons for these seemingly more liberal attitudes towards assisted dying in humans among veterinarians compared to physicians, despite veterinarians' knowledge and experience with animal euthanasia, are not known. This more liberal view among veterinarians may reflect a general trend in society, as acceptance of assisted dying in humans has increased in most Western European countries [33]. Also, the consequences of legalisation of assisted dying in humans would probably affect the role of the physicians very differently compared to veterinarians, as physicians would probably be more directly involved in patient cases than veterinarians would. This would probably also influence the responses given to this questionnaire among the two professional groups.

Working with companion animals was independently associated with positive attitudes towards human euthanasia. As our study is cross-sectional, we do not know if there is a causal relationship in this association. Further, this is a quite novel finding, and qualitative studies could elaborate further on the possible influence of animal euthanasia on veterinarians. Actually, veterinarians in companion animal practice have described viewing euthanasia as an ‘act of compassion’, in which the goal is facilitating a ‘good death’ [34]. This might be in contrast to the work of veterinarians working with production animals, where financial considerations would more often lead to animals being slaughtered and not euthanised. The role that these factors might play in attitudes towards euthanasia in humans needs further exploration.

Veterinarians’ frequency of euthanasing animals was not independently associated with positive attitudes towards human euthanasia. Again, the cross-sectional design hinders any conclusions regarding causality, however, it may suggest that it is not the euthanasia case-load that affects veterinarians’ attitudes, but rather their knowledge and experience of the euthanasia process. Qualitative studies would be a feasible approach to gain a deeper understanding of the possible influence and interplay of different work-related factors on attitudes towards death among veterinarians, and to explore whether veterinarians' view on death is associated with the high suicide rate seen in the profession.

Being younger and being single were also significantly associated with positive attitudes towards euthanasia in humans. This is in line with a recent systematic review that found that being younger and being divorced or being widowed predicted higher endorsement of assisted dying [35].

Performing euthanasia five times or more per week was independently associated with serious suicidal thoughts. A systematic review including 12 papers reported that performing euthanasia may generate traumatic stress and decrease the well-being of animal care workers [36]. Furthermore, several studies have shown that euthanasia is a source of moral and job stress [37–39], while another study did not find a significant relationship between euthanasia and psychological distress or compassion fatigue [40]. Veterinarians have emphasised the lack of sufficient training in euthanasia-related decision-making and euthanasia consultations [34, 38, 40], as they are also responsible for managing animal owners’ grief, guilt, and loss during euthanasia consultations [41]. Our findings suggest that euthanasing animals may be an occupational stressor. This should be further investigated, especially with respect to its potentially contributing to suicide risk. A normalisation or possible habituation process through exposure to animal euthanasia would be in line with one of the three constructs of the interpersonal theory of suicide, namely the acquired capability of suicide [42]. According to the authors, habituation to the pain and fear of suicide may be method-specific and acquired through exposure [42]. The authors exemplified this by presenting the preferred method of suicide in different occupations, i.e., guns in the army, hanging or knots in the navy, and falling or heights in the air force. Such habituation may be especially relevant for veterinarians working in companion animal practice, but further research is needed to elaborate on the role of animal euthanasia in suicide risk among veterinarians. Also, due to the cross-sectional design of our study, we do not know the direction of the association found. Cognitive bias among veterinarians with serious suicidal thoughts cannot be ruled out, possibly leading to an over-reporting of the frequency of animal euthanasia. A previous study from Norway found that veterinarians had a relatively high prevalence of mental health problems in need of treatment [43]. It is known from previous research that depression may lead to an overestimation of negative events, due to cognitive bias [44]. Moreover, positive attitudes towards euthanasia in humans were independently associated with serious suicidal thoughts among veterinarians. Although different measures were used, our findings may support a previous study that found a significant positive correlation between attitudes towards human euthanasia and suicide among veterinarians [7].

Being single was associated with serious suicidal thoughts among veterinarians, consistent with previous findings [24, 27]. Perceived support from colleagues was associated with a reduced likelihood of having serious suicidal thoughts. Traditionally, veterinary work has been an occupation with more professional isolation than other medical professions, especially in rural areas. In fact, professional isolation and a lack of social support have been emphasised as risk factors for suicide among veterinarians [23]. Interventions to strengthen collegiality and facilitate colleague support networks could be an important aim for preventive mental health measures for veterinarians at the organisational level.

Among veterinarians, a register-based study found that when excluding decedents with pentobarbital poisoning, the standardised mortality rate for suicide was not significantly different from that of the general population [2]. Restriction to means has previously been highlighted as an important strategy for suicide prevention [45], and the secure storage of euthanasia solutions has been proposed as a method for addressing veterinarian suicide [46]. Euthanasing animals is a routine occupational task for veterinarians. Their experience in decision-making regarding ‘the right time’ to euthanise may render veterinarians with suicidal thoughts especially vulnerable to suicide by self-poisoning. Indeed, a recent study found that among veterinarians with suicidal ideation in the past week, easy access to lethal medication in their workplace was associated with a six-fold increase in the perceived likelihood of a future suicide attempt, compared to those locking away lethal medication during business hours [47]. Our findings coincide with a recent study that suggests that performing euthanasia affects veterinarians’ attitudes towards suffering, or towards alleviating suffering through death [6]. As self-poisoning is the most commonly used method for suicide, addressing this as an occupational hazard for suicide among veterinarians seems appropriate. Suicide is a multifaceted phenomenon with a complex aetiology, and a dozen risk factors have been described [48]. Therefore, no single factor, such as animal euthanasia, will explain suicidal thoughts or suicide among veterinarians.

To our knowledge, this is the only nationwide study among veterinarians investigating the association between animal euthanasia and serious suicidal thoughts, and between animal euthanasia and attitudes towards euthanasia in humans. A major strength was the study’s high response rate (75%), which made multivariable analyses feasible, while reducing the possible effects of selection and response biases. The questionnaire was quite comprehensive and included several relevant individual and work-related variables for the multivariable statistical models. This is an asset, since we know that explanation of both human attitudes and suicidal thoughts can be multifaceted and complex. An important limitation is the cross-sectional design, restricting conclusions regarding causality. Additionally, this being a self-report study introduces the possibility of recall bias. The generalisability of our results may also be limited. Nevertheless, we believe that our findings are representative of veterinarians in Northern Europe. The study was conducted during the Coronavirus pandemic, which may have affected the results. The survey was planned before the pandemic, and any potential effects (e.g., redundancies and economic effects in the practices) were not accounted for. We did not control for religious views in our study, which could be a possible confounder with regard to attitudes towards assisted dying and serious suicidal thoughts. A challenge with the instrument on attitudes towards assisted dying is that, although we included definitions of the different terms in the questionnaire, the statements and response alternatives leave room for interpretation. For example, ‘short remaining life expectancy’ was not defined, nor were ‘suffering’ or ‘fatal disease’. Also, the response categories were not strictly defined. When using this instrument, we dichotomised the responses as in previous studies, assuming that the respondents were either in favour of or opposed to assisted dying. Specific regulations of assisted dying would probably influence attitudes towards the statements given in the questionnaire. Those responding ‘partially agree’ or ‘partially disagree’ could be receptive to arguments either in favour of or against the legalisation of assisted dying. Therefore, a relatively large proportion of veterinarians are unlikely to hold an extreme attitude in favour of or against legalisation but may be willing to change their minds in different circumstances. This may have reduced the reliability of this measure. When measuring veterinarians’ frequency of euthanasing animals, ‘0’ should have been a separate category, instead of its being grouped with 0–4 per week. Further, we did not define animal euthanasia. It may be that non-justified and absolutely justified animal euthanasia would have yielded different results. This may have reduced the validity of this measure. However, we were able to distinguish between those with a low case-load (0–4/week) and those with a high case-load (5 or more/week). There is a possibility that there is a confounding variable affecting both serious suicidal thoughts and veterinarians’ frequency of euthanasing animals that we have not accounted for in our study. Therefore, a possible association between euthanasing animals and serious suicidal thoughts should be validated in other representative veterinarian samples in the future.

## Conclusions

Veterinarians’ attitudes towards assisted dying in humans did not differ from those of the general population. Veterinarians’ frequency of euthanasing animals was not associated with positive attitudes towards euthanasia in humans. However, veterinarians working with companion animals were more likely to have positive attitudes towards euthanasia in humans. Moreover, euthanising animals five times a week or more was associated with serious suicidal thoughts. Therefore, veterinarians with suicidal thoughts may benefit from not working with euthanasia. Our study is the first nationwide survey investigating the association between animal euthanasia, attitudes to assisted dying and suicidal thoughts among veterinarians. Therefore, our findings should be validated in other veterinary populations. Qualitative studies could further elaborate on the role of animal euthanasia on veterinarians’ suicide risk, as well as on attitudes towards assisted dying and death, and a possible interplay between these factors. For instance, interviews with veterinarians with a history of suicidal thoughts or suicide attempts would be beneficial, to explore whether occupational experience with animal euthanasia affected the suicidal process. The role of euthanasia as an occupational risk for suicide among veterinarians should also be further assessed in prospective studies, both in terms of possibly altered attitudes towards death and professional access to means.

### Supplementary Information

**Additional file 1: Additional Table 1.** Veterinarians’ attitudes toward physician-assisted suicide and euthanasia of humans, gender differences.

**Additional file 2: Additional Table 2.** Cross-table frequency of euthanasia of animals with main field of work.

## Data Availability

Data are available upon reasonable request. Requests should be directed to the corresponding author.
