# Supplementary material for: Euthanasia of animals – association with veterinarians’ suicidal thoughts and attitudes towards assisted dying in humans: a nationwide cross-sectional survey (the NORVET study)
Source: BMC Psychiatry. 2024 Jan 2;24:2. doi: 10.1186/s12888-023-05402-7 (PMC10763301; doi:10.1186/s12888-023-05402-7)
Supplement: Supplementary file 1 — Additional file 1: Additional Table 1. Veterinarians’ attitudes toward physician-assisted suicide and euthanasia of humans, gender differences. [file 12888_2023_5402_MOESM1_ESM.docx]

Additional table 1 - Veterinarians´ attitudes toward physician-assisted suicide and euthanasia of humans, gender differences

| Statement | Strongly agree + Partially agree  n (%) | | Neither agree, nor disagree+ Partially disagree + Strongly disagree  n (%) | | Total, n | χ^2^ |
| --- | --- | --- | --- | --- | --- | --- |
|  | Women | Men | Women | Men |  |  |
| 1. ‘Physician-assisted suicide should be permitted for persons suffering from a fatal disease with a short remaining life expectancy.’ | 1190 (67.8%) | 402 (53.2%) | 566 (32.2%) | 354 (46.8%) | 2512 | 48.5, p<0.001 |
| 1. ‘Euthanasia should be permitted for persons suffering from a fatal disease with a short remaining life expectancy.’ | 1021 (58.2%) | 358 (47.3%) | 734 (41.8%) | 399 (52.7%) | 2512 | 25.3, p<0.001 |
| 1. ‘Assisted dying should be permitted also for persons suffering from an incurable chronic disease, but who are not dying.’ | 816 (46.6%) | 270 (35.8%) | 937 (53.5%) | 484 (64.2%) | 2507 | 24.8, p<0.001 |
| 1. ‘There are cases in which it may be right/morally defensible for the doctor to provide assisted dying, even though it is illegal.’ | 946 (53.9%) | 342 (45.1%) | 808 (46.1%) | 417 (54.9%) | 2513 | 16.7, p<0.001 |
