# Supplementary material for: Euthanasia of animals – association with veterinarians’ suicidal thoughts and attitudes towards assisted dying in humans: a nationwide cross-sectional survey (the NORVET study)
Source: BMC Psychiatry. 2024 Jan 2;24:2. doi: 10.1186/s12888-023-05402-7 (PMC10763301; doi:10.1186/s12888-023-05402-7)
Supplement: Supplementary file 2 — Additional file 2: Additional Table 2. Cross-table frequency of euthanasia of animals with main field of work. [file 12888_2023_5402_MOESM2_ESM.docx]

Additional Table 2 - Cross-table frequency of euthanasia of animals with main field of work

|  | **Frequency of animal euthanasia, n (%)** | |  |
| --- | --- | --- | --- |
| **Main field of work** | **0-4/week** | **5 or more/week** | **Total, n** |
| Companion animals | 732 (92 %) | 61 (8 %) | 793 |
| Production animals | 164 (96 %) | 7 (4 %) | 171 |
| Mixed clinical practice | 243 (92 %) | 20 (8 %) | 263 |
| Equine practice | 97 (99 %) | 1 (1 %) | 98 |
| Aquaculture | 107 (88 %) | 14 (12 %) | 121 |
| Public administration | 400 (99.5%) | 2 (0.5 %) | 402 |
| Academia/research | 202 (100 %) | 0 (0 %) | 202 |
| Other | 240 (96 %) | 10 (4 %) | 250 |
| **Total** | 2185 (95 %) | 115 (5 %) | 2300 |
